# Towards understanding brain-gut-microbiome connections in Alzheimer’s disease

**DOI:** 10.1186/s12918-016-0307-y

**Published:** 2016-08-26

**Authors:** Rong Xu, QuanQiu Wang

**Affiliations:** 1 Department of Epidemiology and Biostatistics, Institute of Computational Biology, School of Medicine, Case Western Reserve University, 2103 Cornell Road, Cleveland, 44106 USA; 2 ThinTek LLC, Palo Alto, 94306 USA

**Keywords:** Systems biology, Network medicine, Alzheimer’s disease, Human gut micriobiome, Disease etiology

## Abstract

**Background:**

Alzheimer’s disease (AD) is complex, with genetic, epigenetic, and environmental factors contributing to disease susceptibility and progression. While significant progress has been made in understanding genetic, molecular, behavioral, and neurological aspects of AD, relatively little is known about which environmental factors are important in AD etiology and how they interact with genetic factors in the development of AD. Here, we propose a data-driven, hypotheses-free computational approach to characterize which and how human gut microbial metabolites, an important modifiable environmental factor, may contribute to various aspects of AD.

**Materials and methods:**

We integrated vast amounts of complex and heterogeneous biomedical data, including disease genetics, chemical genetics, human microbial metabolites, protein-protein interactions, and genetic pathways. We developed a novel network-based approach to model the genetic interactions between all human microbial metabolites and genetic diseases. We identified metabolites that share significant genetic commonality with AD in humans. We developed signal prioritization algorithms to identify the co-regulated genetic pathways underlying the identified AD-metabolite (brain-gut) connections.

**Results:**

We validated our algorithms using known microbial metabolite-AD associations, namely AD-3,4-dihydroxybenzeneacetic acid, AD-mannitol, and AD-succinic acid. Our study provides supporting evidence that human gut microbial metabolites may be an important mechanistic link between environmental exposure and various aspects of AD. We identified metabolites that are significantly associated with various aspects in AD, including AD susceptibility, cognitive decline, biomarkers, age of onset, and the onset of AD. We identified common genetic pathways underlying AD biomarkers and its top one ranked metabolite trimethylamine N-oxide (TMAO), a gut microbial metabolite of dietary meat and fat. These coregulated pathways between TMAO-AD may provide insights into the mechanisms of how dietary meat and fat contribute to AD.

**Conclusions:**

Employing an integrated computational approach, we provide intriguing and supporting evidence for a role of microbial metabolites, an important modifiable environmental factor, in AD etiology. Our study provides the foundations for subsequent hypothesis-driven biological and clinical studies of brain-gut-environment interactions in AD.

## Background

Human gut microbiota (>10^14^ microbial cells comprising about 1000 different species) are important modifiable environmental factors that we are exposed to continuously [1]. These microbiota exist in a symbiotic relationship with a human host by metabolizing compounds that humans are unable to utilize and by controlling the immune balance of the human body [2]. Accumulating clinical and biomedical evidence indicates that gut microbiota and their metabolites influence brain function and behavior in a range of central nervous system (CNS) disorders, including depression, cognitive decline, autism, and multiple sclerosis [3].

Human gut microbiota contribute to brain function, not only via neural, humoral, immune pathways, but also via the cumulative effects of microbial metabolites [3]. Human metabolism encompasses a combination of microbial and human enzyme activities [4]. Undigested dietary components are fermented by microbiota to produce a wide array of metabolites such as bile acids, choline and short-chain fatty acids (SCFAs) that are essential for health [1]. It has become increasingly clear that metabolite activities of gut microbiota provide a mechanistic connection between environmental factors and brain function and behavior [3, 5].

Although the link between microbial metabolism and brain has been recognized, the complex relationships between microbial metabolites and AD remain uncharacterized; the mechanisms underlying how microbial metabolites interact with AD genetics in promoting or protecting against AD remain unknown. Computational approaches have been widely used in biomedical fields, including drug discovery [6–10] and disease genetics prediction [11–13]. In one of our recent studies, we developed a hypothesis-driven genome-wide systems approach to reveal the strong mechanistic links between colorectal cancer and trimethylamine N-oxide (TMAO), a gut microbial metabolite of dietary meat and fat [14]. To date, however, computational approaches to systematically characterizing and understanding the complex host genome-microbiome metabolism interactions in AD have not been undertaken. Here, we propose a comprehensive, data-driven, hypotheses-freeb computational approach to characterize which and how gut microbial metabolites interact with AD genetics in humans.

## Data sets

**Fig. 1:**
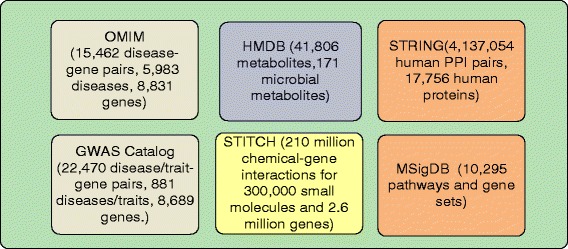
Datasets used in this study

We used the publicly available databases of human metabolome, disease genetics, chemical genetics and protein functional interactions, and signaling pathways for our task of characterizing and understanding human gut microbial metabolites that are genetically related to AD (Fig. 1).

### The Human Metabolome Database (HMDB)

We used the HMDB to obtain a list of metabolites produced by human gut microbiota. The HMDB contains detailed information about small molecule metabolites found in the human body [15]. The database contains 41,806 metabolites, among which 171 metabolites originated in human microbial metabolism.

### Chemical genetics data

We used STITCH (Search Tool for Interactions of Chemicals) database [16] to obtain metabolite-gene associations for the 171 microbial metabolites from the HMDB. STITCH is a database of known and predicted interactions of chemicals and proteins supported by evidence derived from experiments, curated databases, and published literature [16]. STITCH contains data on the interactions between 300,000 small molecules and 2.6 million proteins from 1133 organisms, each interaction being associated with a score measuring the evidence of the association. In this study, we used chemical-gene associations in humans.

### Disease genetics data

We used two complementary disease genetics databases to obtain disease-gene associations. The first data resource is the Catalog of Published Genome-Wide Association Studies (GWAS catalog) from the US National Human Genome Research Institute (NHGRI) [17]. The GWAS catalog is an exhaustive source containing descriptions of disease/trait-associated single nucleotide polymorphisms (SNPs) from published GWAS data. Currently, the GWAS catalog contains 22,470 disease/trait-gene pairs, representing 8,689 genes and 881 common complex diseases/traits, including multiple aspects of AD (“cognitive decline,” “biomarkers,” “age of onset,” and “late onset”).

The second resource of disease genetics is the Online Mendelian Inheritance in Man database (OMIM), currently the most comprehensive source of disease genetics for Mendelian disorders [18]. OMIM contains both rare Mendelian genetic disorders and mutations that can cause susceptibility to multifactorial disorders. Currently, OMIM includes 15,462 disease-gene pairs for 8,831 genes and 5,983 diseases, including “susceptibility to AD.”

### Protein-protein interaction data

We used the functional protein-protein interaction (PPI) data from the STRING (Search Tool for the Retrieval of Interacting Genes/Proteins) database to model the genetic interactions between metabolites and diseases. STRING is a comprehensive functional PPI database and contains 4,137,054 PPI pairs in human, representing 17,756 proteins/genes [19].

### Genetic pathway data

We used the rich pathway information from the Molecular Signatures Database (MSigDB) [20] to identify the interplaying pathways underlying identified microbial metabolites and AD. Currently, MSigDB contains 10,295 annotated pathways and gene sets collected from various sources such as online pathway databases, literature, knowledge of domain experts, expression signatures of genetic and chemical perturbations, and cell states and perturbations within the immune system.

## Methods

### Find microbial metabolites that are significantly associated with AD

#### Construct genetic disease networks (GDNs)

**Fig. 2:**
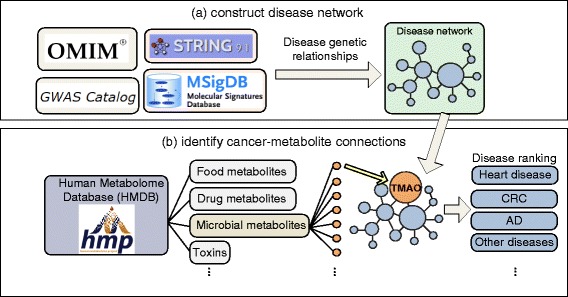
Model the genetic interactions between microbial metabolites and diseases by constructing a metabolite-disease genetic interaction network (mGDN) (**a**) and prioritizing diseases, including AD, for each metabolite (**b**)

We constructed two genetic disease networks using 22,470 disease/trait-gene pairs from the GWAS catalog and 15,462 disease-gene pairs from OMIM, respectively. On each network, two diseases are connected if they share genes. The edge weights on the networks were determined by cosine similarities [21] of disease-associated genes. Since some diseases do not share genes directly but their associated genes interact or participate in the same pathways, we also investigated an alternative approach to connect diseases on the networks: two diseases were connected if their associated genes (proteins) interact or participate in the same pathways. We used functional protein-protein interaction data from the STRING database to model the genetic interactions between metabolites and diseases (Fig. 2a).

#### Model genetic interactions between microbial metabolites and diseases

For each of the 171 metabolites, we modeled genetic interactions between the metabolite and all diseases by inserting a node representing the metabolite into GDNs (Fig. 2b). On the transformed metabolite-disease genetic network (mGDN), a metabolite node is connected to a disease node if the metabolite-associated genes overlap with disease-associated genes. Similar to the original GDN construction, the edge weights between the inserted metabolite and disease nodes were determined by the cosine similarity between the metabolite- and the disease-associated genes. We generated 1000 random mGDNs by randomly shuffling the edges of the real mGDN. Random mGDNs were used to assess the significance of the associations between metabolites and AD.

#### Find AD-associated metabolites

We applied the network-based ranking algorithms that we recently developed [8–10, 12–14] to prioritize diseases that are genetically related to each of the 171 metabolites. The output of this network-based ranking algorithm is a list of ranked diseases (AD and other diseases) for each of the 171 microbial metabolites (Fig. 2b).

#### Establish statistical significance of metabolite-AD associations

For each metabolite (e.g. TMAO, butyrate, acetate), we obtained a ranked list of diseases from the real mGDN and 1000 ranked lists of diseases from the 1000 random mGDNs. We compared the ranking of AD among diseases derived from the real mGDN (i.e. that AD ranked among the top 1.25 % for TMAO) to those from random mGDNs (that AD ranked among the top 44 % on average for TMAO) and performed a t-test to assess statistical significance.

#### Algorithm validation

In this study, we performed the following evaluations: (1) we tested our algorithm using known AD-associated microbial metabolites from HMDB: *3,4-dihydroxybenzeneacetic acid*, *mannitol*, and *succinic acid*; (2) we evaluated top 20 associations by performing literature search; (3) we tested if the same observations were seen when two complementary disease genetics data resources were used: the GWAS catalog and the OMIM database; (4) we tested if the same observations were seen across multiple traits of AD, such as “susceptibility,” “cognitive decline,” “biomarkers,” “age of onset,” and “late onset;” and (5) we tested if the metabolites are also involved in AD-associated genetic pathways (described later).

### Identify signaling pathways that may be co-regulated by AD and metabolites

**Fig. 3:**
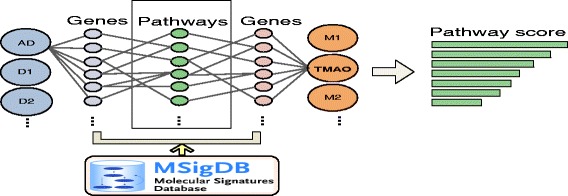
Find shared genetic pathways between AD and its associated metabolites

To better understand the molecular mechanisms underlying identified AD-metabolite connections, we investigated genetic pathways that may be co-regulated by both the metabolites and AD. We selected one specific metabolite, namely trimethylamine n-oxide (TMAO), for our analysis. In our study, TMAO is most significantly associated with AD biomarkers. We retrieved a total of 54 TMAO-associated human genes from STITCH. We obtained a total of 26 genes associated with AD biomarkers from the GWAS catalog. For each TMAO- or AD-associated gene, we retrieved its corresponding genetic pathways from MSigDB. We then obtained a set of genetic pathways for TMAO and a set of AD-associated genetic pathways. We intersected TMAO- and AD-associated pathways to identify the shared pathways. These shared pathways were then prioritized on the basis of their relevance to both TMAO and AD. The ranking score for a given common pathway is defined as: $R_{common\_pathway}=\sum \limits _{i=1}^{n} G_{i}\sum \limits _{j=1}^{m} G_{j}$, where *G*_*i*_ is a TMAO-associated gene in the pathway and *G*_*j*_ is a AD-associated gene in the same pathway. The intuition is that a pathway that contains both many TMAO-associated genes and many AD-associated genes will rank higher than a pathway that contains fewer such genes (Fig. 3).

## Results

### Known AD-associated metabolites ranked highly

We evaluated our algorithm using three known metabolites: mannitol, succinate, and 3,4-dihydroxyphenylacetaldehyde (DOPAL). Mannitol is a sugar alcohol. Studies indicate that mannitol is associated with AD [22] and other diseases including AIDS, cytochrome C oxidase deficiency, lung cancer, and ribose-5-phosphate isomerase deficiency. Succinic acid is a dicarboxylic acid and a component of the citric acid cycle electron transfer chain in the mitochondria. Studies show that succinic acid is associated with AD [23] and other human mitochondrial disease such as Hungtinton disease. DOPAC is a phenolic acid and a neuronal metabolite of dopamine (DA). Studies have demonstrated that DA-derived aldehyde is a reactive electrophile and toxic to dopaminergic cells. DOPAC is associated with AD [24] and other neurological disorders including Parkinson’s disease, Encephalitis [25].

Our study demonstrate that mannitol is significantly associated with multiple traits of AD including “cognitive decline,” “biomarkers,” “late onset,” and “susceptibility” (Table 1). Succinic acid is significantly associated with both ‘cognitive decline’ and ‘susceptibility’ in AD. DOPAC is significantly associated with ‘biomarkers’, ‘age of onset’ and ‘susceptibility’ in AD (Table 1). In summary, previous biomedical studies demonstrated altered levels of these three metabolites in AD patients [22–24]. Our study provides additional evidence that these microbial metabolites may be mechanically linked to AD genetics through shared genes or genetic pathways.

**Table 1:** Three known AD-associated metabolites ranked highly

Metabolite	AD types	Enrichment	*P* value
Mannitol	AD cognitive decline	353.4 %	2.91E-28
	AD biomarkers	201.5 %	1.16E-19
	AD (late onset)	54.8 %	6.23E-13
	AD, susceptibility to	8957.1 %	2.34E-32
Succinic acid	AD cognitive decline	459.5 %	2.37E-31
	AD, susceptibility to	1662.5 %	1.25E-30
3,4-dihydroxybenzeneacetic acid (DOPAC)	AD biomarkers	262.7 %	9.40E-26
	AD (age of onset)	47.2 %	1.75E-8
	AD, susceptibility to	1602.5 %	1.49E-30

### Microbial metabolites that are significantly associated with AD

**Fig. 4:**
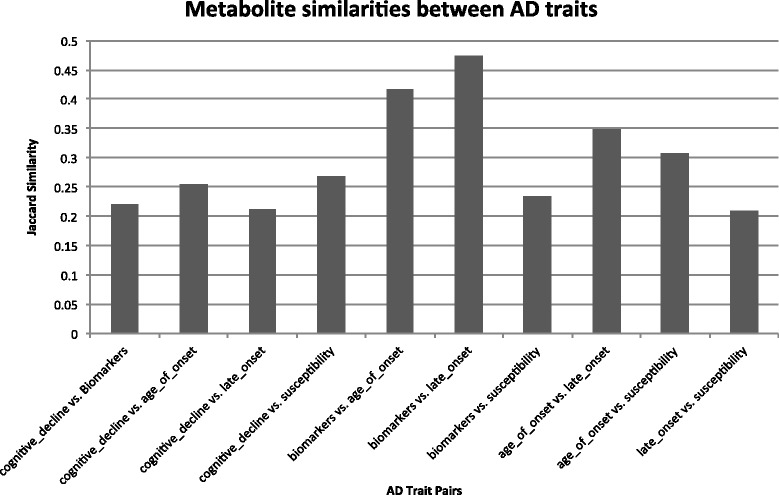
Jaccard similarities of metabolites that are significantly associated with two different traits of AD (*cognitive decline, biomarkers, age of onset, late onset, susceptibility*)

We identified 56 metabolites significantly associated with “cognitive decline” in AD, 62 with “biomarkers,” 59 with “age of onset” in AD, 55 with “late onset” of AD, and 45 with AD susceptibility. As shown in Fig. 4, the metabolites associated with one trait/aspect of AD are quite different from ones associated with the other aspects of AD. For example, the Jaccard similarity, defined as the size of the intersection divided by the size of the union of two sets [21], between cognitive decline in AD (56 metabolites) and AD biomarkers (62 metabolites) is 0.22, which is much higher than random exception ((56/171)*(62/171)=11.8 %). The two highest profile similarities are between AD biomarkers and age of onset in AD (similarity = 0.42), and between AD biomarkers and late onset of AD (similarity = 0.47). Given that metabolite similarities between different aspects of AD are significantly higher than random expectation, our results provide an intriguing hypothesis that gut microbial metabolites may be one of mechanistic links between different aspects of AD.

For the trait “cognitive decline” in AD, we manually evaluated its top 20 metabolites by searching literature for supporting evidence. Table 2 shows top 20 metabolites along with their enrichments over random, *p* value as well as literature evidence supporting their roles in AD. The three known AD-associated metabolites (mannitol, succinic acid, and DOPAC) were among top 20 metabolites.

**Table 2:** Top 20 metabolites that are significantly associated with cognitive decline in AD

Metabolite	Enrichment	*P* value	Literature
	fold		
d-proline	28.7	1.50E-39	[26]
1,2,3-propanetricarboxylic acid	19.1	8.45E-39	
5-aminopentanoic acid	12.1	1.80E-37	
chenodeoxycholic acid	8.5	6.29E-36	[27]
glycine conjugate			
cadaverine	7.66	2.42E-35	[28]
benzoyl-coa	7.66	2.42E-35	
diaminopimelic acid	5.9	1.45E-33	
putrescine	4.9	5.85E-32	[28]
trehalose	4.7	1.49E-31	
**succinic acid**	4.6	2.37E-31	[23]
5-methylthioribulose 1-phosphate	4.4	6.05E-31	
pyrrolidine	4.2	2.49E-30	[32]
citramalic acid	4.0	6.42E-30	
trans-ferulic acid	3.8	4.30E-29	[29–31]
**mannitol**	3.5	2.91E-28	[22]
**4-hydroxybenzoic acid (DOPAC)**	3.6	2.91E-28	[24]
d-glutamic acid	3.4	7.60E-28	[35]
melibiose	3.3	1.99E-27	
5alpha-cholestanol	3.3	3.24E-27	[33, 34]
1-butanol	3.0	9.63E-26	

The three known metabolites (mannitol, succinic acid, and DOPAC) are highlighted

D-proline ranked at top one. Studies show that a bis(d-proline) compound, (R)-1-[6-[(R)-2-carboxy-pyrrolidin-1-yl]-6-oxo-hexanoyl]pyrrolidine-2-carboxylic acid, depleted circulating serum amyloid P component from cerebrospinal fluid in AD [26]. Our results indicate that targeting bacteria producing d-proline may provide an attractive alternative therapeutic approach in removing amyloids from brain, therefore reversing or inhibiting cognitive decline in AD.

Several secondary bile acids ranked highly, including chenodeoxycholic acid glycine conjugate (top 4), taurochenodesoxycholic acid (top 24), and taurodeoxycholic acid (top 26). Secondary bile acids are potent inhibitors of apoptosis in different cell types. The potential role of apoptosis in Alzheimer’s disease (AD) has been an area of intense research in recent years. Studies provide evidence for the anti-apoptotic role of bile acids in experimental AD [27].

Both cadaverine and putrescine ranked highly. Cadaverine and putrescine are polyamine, which are known to be closely related with cell growth, cell proliferation, and synthesis of proteins and nucleic acids. The neurotoxic amyloid ?-peptide in AD is known to up-regulate polyamine metabolism by increasing ornithine decarboxylase activity and polyamine uptake by initiating free radical damage. Polyamines play an important role in response to neurodegenerative conditions. Altered levels of polyamines have been found in tissue, hair and body fluids of patients with neuromuscular diseases and neurodegenerative conditions [28].

Trans-ferulic acid ranked at top 14. Trans-ferulic acid is one of the most abundant phenolic acids in fruit and vegetables and a potent antioxidant. Free-radicals derived from mitochondrial dysfunction and from the cyclooxygenase enzyme activity play a role in oxidative damage of brain. Food rich in ferulic acid and other the antioxidant is considered a nutritional approach to reduce oxidative damage and amyloid pathology in AD [29–31].

Pyrrolidine ranked at top 12. Pyrrolidine dithiocarbamate (PDTC) is a nuclear factor- *κ*B (NF- *κ*B) inhibitor, antioxidant and antiinflammatory agent. PDTC activates Akt and improves spatial learning in mouse model of AD [32].

Recent epidemiological, clinical, and experimental data suggest that cholesterol may play a role in AD pathogenesis and plaque formation. Cholesterolemia is involved in the development of amyloid in AD. Recent work demonstrated that diet-induced hypercholesterolemia resulted in dramatic acceleration of the neuropathological and biochemical changes in the transgenic mice [33, 34].

D-glutamic acid ranked at top 17. Glutamate is the major fast excitatory neurotransmitter and is involved in almost all CNS functions. Severe disturbances in glutamate neurotransmission has been linked with the pathophysiological processes underlying AD [35].

### Genetic pathways that may be co-regulated by TMAO and AD

TMAO ranked at top one for AD biomarkers. Recent studies have shown a mechanistic link between TMAO, a gut microbial metabolite of dietary meat and fat, and risk of CVDs, and established an obligatory role of gut microbiota in the generation of proatherosclerotic TMAO from dietary L-carnitine and phosphatidylcholine, abundantly present in red meat and dietary fat, respectively [36–39]. Our results showing that TMAO is highly associated with AD is consistent with epidemiological evidence that western diet rich in high fat is associated with AD [40–43]. Multiple cohort studies and large randomized trials have suggested that Mediterranean diet, which is low in red meat and high in fruits, vegetables, whole grains, beans, nuts, and seeds improves cardiovascular outcomes, including stroke, and these effects may directly or indirectly promote lower dementia risk [44, 45]. Our study demonstrate that TMAO is genetically associated with AD and this finding is consistent with observed correlations between AD and CVD dietary risk factors and the mechanistic links between TMAO and CVD pathogenesis.

We investigated the potential genetic pathways underlying the strong association between TMAO and AD. We identified a total of 27 genetic pathways associated with AD biomarkers, and a total of 171 pathways associated with TMAO-related genes. Among these pathways, 9 pathways are involved in both AD and TMAO, including pathways related to “Alzheimer’s disease,” “Axon guidance,” “immune systems,” “neuron signaling,” and “lipid and protein metabolism”. Table 3 shows top 9 pathways for AD, TMAO, and both. These pathways may provide insights into the diet-gut-microbiome-brain interactions. The fact that AD is highly associated with TMAO provides intriguing supporting evidence for a role of diet and microbial metabolites in AD etiology.

**Table 3:** Top nine ranked genetic pathways associated with AD (biomarkers), TMAO and both

AD	TMAO	AD ∩TMAO
(27 pathways)	(171 pathways)	(9 pathways)
Lipoprotein metabolism	Cysteine and methionine metabolism	Metabolism of proteins
Metabolism of lipids and lipoproteins	Pyruvate metabolism	Immune system
Axon guidance	Glycolysis / Gluconeogenesis	Adaptive immune system
Alzheimer’s disease	Propanate metabolism	Alzheimer’s disease
Amyotrophic lateral sclerosis (ALS)	Transcription	Axon guidance
Mitochondrial Protein Import	Amyloids	Amyotrophic lateral sclerosis (ALS)
Cell junction organization	The citric acid (TCA) cycle and respiratory electron transport	EPHA forward signaling
HDL-mediated lipid transport	RNA Polymerase I, RNA Polymerase III, and Mitochondrial Transcription	EPHB forward signaling
Lipid digestion, mobilization, and transport	Splicesome	Metabolism of lipids and lipoproteins

## Discussion and conclusions

Alzheimer’s disease is complex, with genetic, epigenetic, and environmental factors contributing to disease susceptibility and progression. Accumulating clinical and biomedical evidence indicates that gut microbiota and their metabolites influence brain function and behavior in a range of central nervous system (CNS) disorders. Employing an integrated computational approach, we provide intriguing and supporting evidence for a role of microbial7metabolites in AD etiology. Our algorithm is highly dynamic and flexible and additional disease genetic data can be easily incorporated. Our study could serve as a starting point for others to conduct hypothesis-driven functional studies of gut-brain-environment interactions in AD and other diseases. In summary, the identification of microbial metabolites and the understanding of their role as key mediators through which these bacteria promote/protect against AD may provide insight into the basic mechanisms of AD etiology, facilitate our understanding of the complex host genome-microbiome interactions in AD pathogenesis, and enable/activate new possibilities for AD diagnosis, prevention, and treatment.
